# Improving shared decision-making in vascular surgery by implementing decision support tools: study protocol for the stepped-wedge cluster-randomised OVIDIUS trial

**DOI:** 10.1186/s12911-020-01186-y

**Published:** 2020-07-23

**Authors:** S. M. L. de Mik, F. E. Stubenrouch, D. A. Legemate, R. Balm, D. T. Ubbink

**Affiliations:** Department of Surgery, Amsterdam UMC, University of Amsterdam, Amsterdam Cardiovascular Sciences, Meibergdreef 9, 1105AZ Amsterdam, The Netherlands

**Keywords:** Shared decision-making, Decision support tools, Vascular surgical procedures, Randomised controlled trial, Study protocol

## Abstract

**Background:**

Shared decision-making improves the quality of patient care. Unfortunately, shared decision-making is not yet common practice among vascular surgeons. Thus, decision support tools were developed to assist vascular surgeons and their patients in using shared decision-making. This trial aims to evaluate the effectiveness and implementation of decision support tools to improve shared decision-making during vascular surgical consultations in which a treatment decision is to be made.

**Methods:**

The study design is a multicentre stepped-wedge cluster-randomised trial. Eligible patients are adult patients, visiting the outpatient clinic of a participating medical centre for whom several treatment options are feasible and who face a primary treatment decision for their abdominal aortic aneurysm, carotid artery disease, intermittent claudication, or varicose veins. Patients and vascular surgeons in the intervention group receive decision support tools that may help them adopt shared decision-making when making the final treatment decision. These decision support tools are decision aids, consultation cards, decision cards, and a practical training. Decision aids are informative websites that help patients become more aware of the pros and cons of the treatment options and their preferences regarding the treatment choice. Consultation cards with text or decision cards with images are used by vascular surgeons during consultation to determine which aspect of a treatment is most important to their patient. In the training vascular surgeons can practice shared decision-making with a patient actor, guided by a medical psychologist. This trial aims to include 502 vascular surgical patients to achieve a clinically relevant improvement in shared decision-making of 10 out of 100 points, using the 5-item OPTION instrument to score the audio-recordings of consultations.

**Discussion:**

In the OVIDIUS trial the available decision support tools for vascular surgical patients are implemented in clinical practice. We will evaluate whether these tools actually improve shared decision-making in the consultation room. The stepped-wedge cluster-randomised study design will ensure that at the end of the study all participating centres have implemented at least some of the decision support tools and thereby a certain level of shared decision-making.

**Trial registration:**

Netherlands Trial Registry, NTR6487. Registered 7 June 2017. URL: http://www.trialregister.nl/trialreg/admin/rctview.asp?TC=6487

## Background

Physicians aim to offer the best quality of care to their patients. In recent years it has been acknowledged that the incorporation of the patients’ preferences, known as shared decision-making (SDM), improves quality of care by enhancing patient satisfaction and therapy adherence [1, 2]. SDM also decreases the number of patients who opt for (major) invasive treatment or who undergo undesired care without adverse effects on health outcomes [1–5].

SDM may especially benefit patients in vascular surgery, because for many patients more than one treatment option is feasible, for example a conservative, endovascular or open surgical treatment, each with their own beneficial and potential harmful effects [6]. It is therefore essential that vascular surgeons are aware of how the patient weighs the benefits and harms of the available options. Unfortunately, studies show that in the Netherlands, the level of SDM is limited among vascular surgeons and that patients are informed inconsistently about their disease and treatment options [7, 8].

In order to improve SDM, a set of decision support tools (DSTs) has been developed for both vascular surgeons and patients. When developed and applied correctly, DSTs improve disease-specific knowledge and, more importantly, SDM in the consultation room [1, 5, 9–12].

DSTs have been developed for Abdominal Aortic Aneurysm (AAA), Carotid Artery Disease (CAD), Intermittent Claudication (IC) and Varicose Veins (VV). These DSTs are designed according to international standards [13] and consist of decision aids, consultation cards, decision cards, and a practical training in SDM for vascular surgeons, physician assistants, and nurse practitioners.

### Objectives

The objective of this trial is to evaluate the effectiveness and implementation of DSTs at the individual patient level to improve SDM during vascular surgical consultations in which a treatment decision is to be made for patients with an abdominal aortic aneurysm, carotid artery disease, intermittent claudication and varicose veins.

## Methods

The study protocol is designed according to the Standard Protocol Items: Recommendations for Interventional Trials (SPIRIT) statement and the CONsolidated Standards of Reporting Trials (CONSORT) extension for Cluster Trials [14, 15]**.** A filled out SPIRIT checklist regarding this trial is added as a supplementary file. The trial was registered in The Netherlands National Trial Registry as NTR6487, available at www.trialregister.nl.

### Trial design

The Operative Vascular Intervention Decision-making Improvement Using Sdm-tools (OVIDIUS) trial design is a 15-center stepped-wedge cluster randomised trial in the Netherlands, as shown in Table 1. Each cluster consists of three participating medical centres. The reasons for choosing this design is in the first place that it allows the evaluation of outcomes before and after introduction of the DSTs in the individual centres and limits the influence of any intercurrent changes in protocols on the clinical outcomes. Second, all participating centres will eventually have implemented at least some of the DSTs and thereby a certain level of SDM.

**Table 1:**
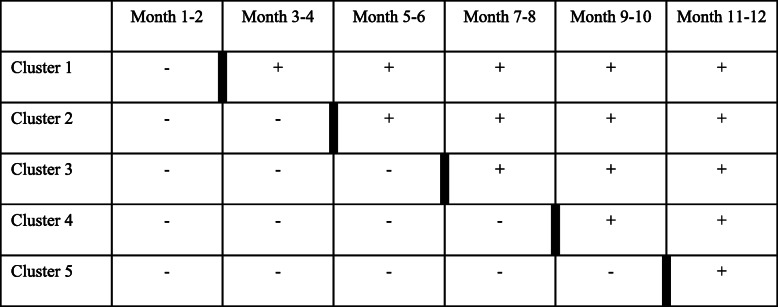
Multicentre stepped-wedge cluster-randomised design

-: Before introduction of decision support tools

+: After introduction of decision support tools

: Introduction of decision support tools/evaluation moment

### Trial setting

Participating centres are located throughout the Netherlands and must provide care for at least one of the four vascular diseases for which the DSTs have been developed. The list of participating medical centres will be published alongside the trial results and is available upon request by emailing the corresponding author. Patients are to be included between January 1, 2018 and June 30, 2019.

### Eligibility criteria

Eligible patients are adults visiting the outpatient clinic of a participating centre who need to decide on a primary treatment for their AAA, CAS, IC or VV. These patients must be eligible for more than one treatment option. Table 2 shows a more detailed overview of the study’s inclusion and exclusion criteria.

**Table 2:** Eligibility criteria

Inclusion criteria	Exclusion criteria
Age ≥ 18 years	Patients requiring emergency surgery
> 1 feasible treatment options	Life expectancy less than 1 year
(Newly) diagnosed with an asymptomatic AAA that has grown to ≥5 cm in women or ≥ 5.5 cm in men	ASA-IV patients
Newly diagnosed with symptomatic CAD with a > 70% stenosis within 6 months since the onset of symptoms, or > 50% in men diagnosed within 12 weeks since the onset of symptoms [16]	Insufficient understanding of the Dutch language
(Newly) diagnosed with invalidating IC (Fontaine II) Considering treatment for VV	Cognitively unable to complete Dutch questionnaires
Willing to sign an informed consent form	

### Interventions

The intervention comprises a set of DSTs, developed to help both vascular surgeons and patients to improve SDM. Use of the DSTs is compared to standard care at the level of individual participants. Standard care may include informative leaflets or websites that participating medical centres already provide to their patients.

The DSTs studied here are decision aids, consultation cards, decision cards and a practical training. The patient advocacy society (Heart and Vascular Group) and the Dutch professional society (Dutch Society for Vascular Surgery) provided intellectual support for the development of the DSTs. The Netherlands Organisation for Health Research and Development provided financial support (ZonMw, grant 516,022,506). The participating centres may decide which combination of DST they prefer to use.

*Decision aids* are validated web-based applications that provide patients with information about their disease and treatment options. In addition, it has an interactive section in which the patient is encouraged to consider what he or she believes is important when deciding on a treatment strategy [9, 17]. Patients receive the decision aid prior to the decision-making consultation via a personalised web link. The researchers automatically receive the answers given by patients in the decision aid regarding their disease-specific knowledge and treatment preferences. The following link provides access to the English version of the Dutch decision aid used in this study for patients with an AAA: https://sdmstaging.medify.eu/surgery1/index_da-aortic-aneurysm_en.html

More information about other available decision aids is provided at the website of the Ottawa Hospital Research Institute [18].

*Consultation cards* are validated tools, also known as Option Grids™ [19]. These are A4-sized paper sheets showing questions -with their answers- that patients most frequently ask about the treatment options, presented in a table format. Vascular surgeon and patient discuss the consultation cards during the consultation. The order in which the patient wants to discuss the questions provides insight into the aspects patients find relevant to them personally when deciding on a treatment strategy [11]. Table 3 shows the consultation card used in this study by vascular surgeons for patients with symptoms of intermittent claudication. More information about other available decision aids is provided at the website of the Dartmouth Institute [20].

**Table 3:** Example of a consultation card for patients with intermittent claudication. Treatment options for intermittent claudication. Use this consultation card if you want to talk to your health care professional about how to treat your blocked or narrowed leg arteries (medical term: ‘intermittent claudication’). This way you can decide with your doctor which option is best for you

Frequently asked questions	(Supervised) exercise therapy	Endovascular treatment (with or without stenting)	Surgery (Endarterectomy or bypass)
What does the treatment entail?	You will exercise on a treadmill (supervised by a physical therapist) to increase your overall and pain-free walking distance. You also receive weight training exercises to practice at home.	A wire is inserted into the artery in your groin. Attached to this wire is a balloon. The balloon is inflated to reduce the narrowing. Sometimes, a tube is left behind to keep the artery open.	▪ With an ‘endarterectomy’ the artery is opened and the narrowing surgically removed. ▪ With a ‘bypass’ either one of your own veins or an artificial tube is used to bypass the narrowed artery.
	You will also continue to take medication to prevent a heart attack or stroke.	You will also continue to take medication to prevent a heart attack or stroke.	You will also continue to take medication to prevent a heart attack or stroke.
What are the benefits of this treatment?	Your general condition will improve due to exercise therapy. There are no treatment risks.	Your complaints will be less immediately after endovascular treatment.	Your complaints will be less immediately after surgery.
What are the main risks associated with the treatment?	You will not have an immediate effect of exercise therapy. It takes about 3 to 6 months before you experience improvement. Some patients will not be able to walk completely pain-free after exercise therapy.	You may suffer from a hematoma (bruise), a reduced kidney function, or the endovascular treatment might even worsen your complaints.	You may suffer from a hematoma (bruise), a wound infection, or the surgery might even worsen your complaints.
What is the effect of the treatment?	After 6 months of exercise therapy, patients like yourself are able to walk twice as far as before the exercise therapy.	Two years after endovascular treatment, the walking distance is about the same as after exercise therapy only.	Two years after surgery, the walking distance is about the same as after exercise therapy only.
Will I receive anaesthesia?	No.	Yes; local anaesthesia.	Yes; general or local anaesthesia.
How long do I stay in the hospital?	No hospital stay.	Usually 1 to 2 days.	Usually 1 week.
What is the risk of losing my leg (amputation)?	1 to 3 of 100 people (1–3%) with intermittent claudication have an amputation within 10 years.	1 to 3 of 100 people (1–3%) with intermittent claudication have an amputation within 10 years.	1 to 3 of 100 people (1–3%) with intermittent claudication have an amputation within 10 years.
What more should I need to know about intermittent claudication?	Exercise therapy does not prevent worsening of the disease. In case of insufficient results, endovascular treatment and surgery are still possible.	Endovascular treatment does not prevent worsening of the disease. Even if you have undergone this treatment, exercise therapy will remain helpful.	Surgery does not prevent worsening of the disease. Even if you have undergone surgery, exercise therapy will remain helpful.
What can I do myself?	The most important things you can do to prevent worsening of the disease is to quit smoking, take plenty of exercise, healthy food, and live a healthy life.	The most important things you can do to prevent worsening of the disease is to quit smoking, take plenty of exercise, healthy food, and live a healthy life.	The most important things you can do to prevent worsening of the disease is to quit smoking, take plenty of exercise, healthy food, and live a healthy life.

Authors: Department of Vascular Surgery Amsterdam UMC location AMC, Heart and Vascular Group, Dutch Society for Vascular Surgery

Based on: most recently available literature

Publication date: May 16, 2017

**Fig. 1:**
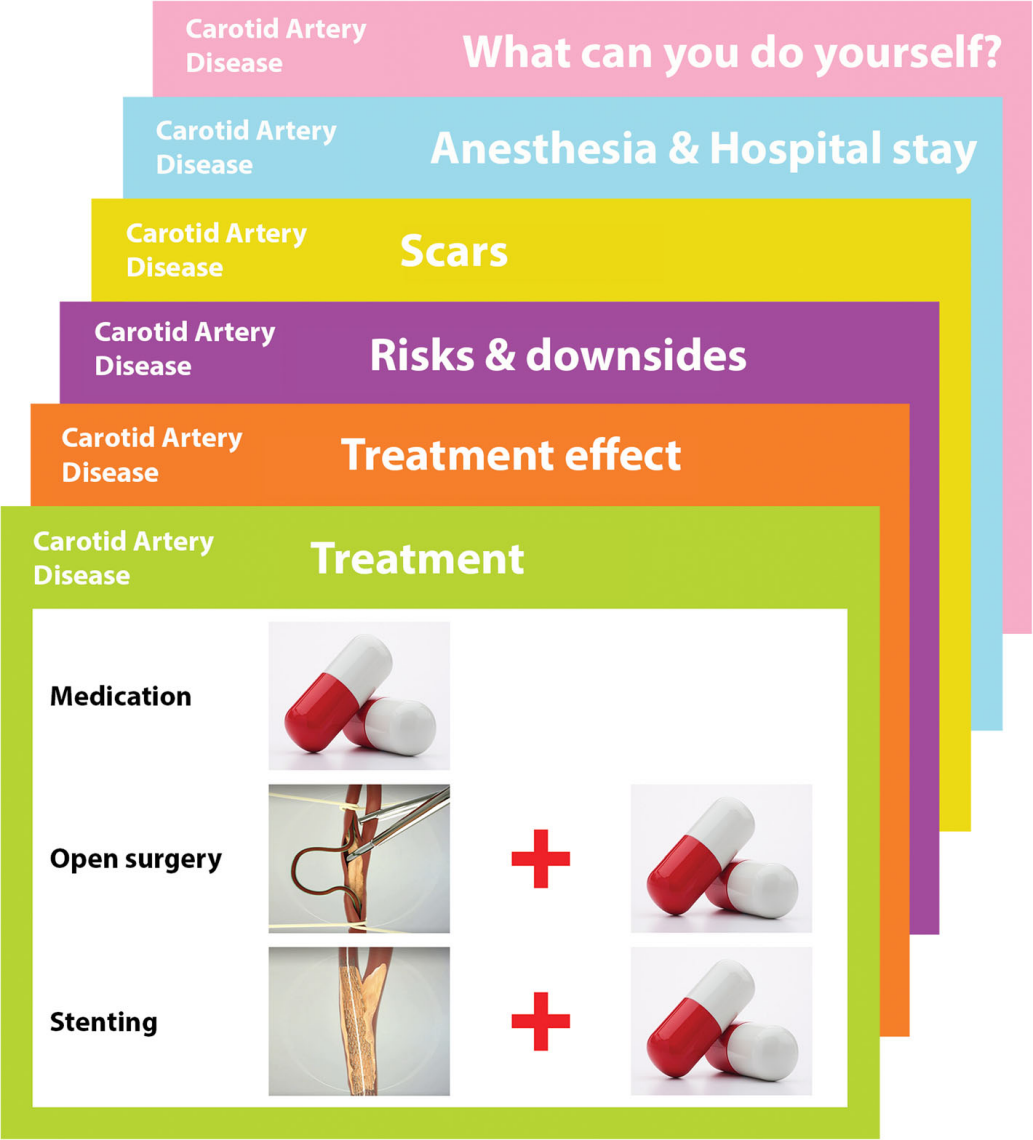
Carotid artery disease decision cards

*Decision cards* are tools designed with the same purpose as consultation cards. Here each question with its answer is presented on a different card. The answers are provided in the form of images, which is supposed to have a beneficial effect on doctor-patient interaction as it leaves room for tailor-made information based on patient comorbidity or hospital performance [10]. Fig. 1 shows the decision cards addressing symptomatic carotid artery disease used in this study by vascular surgeons with their patients. More information about how to use decision cards is provided at the website of the Mayo Clinic Shared Decision Making National Resource Center [21].

*The practical training* is offered to all vascular surgeons, physician assistants and nurse practitioners in the participating centres. The training allows participants to practice the three important steps of SDM, which are the ‘team talk’, ‘option talk’ and ‘decision talk’ [22]. The participants practice these steps with a patient actor under the guidance of a medical psychologist [12, 22]. The practical training takes place just before the vascular surgeons start using the DSTs in their centre.

### Outcomes

The primary outcome is the level of SDM during the consultation as scored with the 5-item Observing patient involvement (OPTION) instrument [23]. The 5-item OPTION instrument allows researchers to objectively assess the level of patient involvement in the decision-making process as scored from audio-recordings of the consultations [24]. If the vascular surgeon and patient need more than one consultation to reach a treatment decision, all consultations are audio-recorded and scored as one consultation. Afterwards, two researchers independently score the five OPTION-items on a five-point scale. The cumulative OPTION-score is expressed on a 100-point scale.

Baseline characteristics, i.e. age, gender, diagnosis, highest level of education, employment status, social status, and ethnicity, are collected from the patients using a questionnaire before consultation.

Secondary outcomes are patients’ disease specific knowledge, decisional conflict, quality of life, and SDM as perceived by patients, SDM as perceived by vascular surgeons, the treatment decided upon, the implementation of DSTs, and process measures.

The patients’ disease-specific knowledge is scored directly after the consultation. The questions test whether patients correctly understood the information presented in the decision aid or received during the consultation.

Decisional conflict in patients is scored directly after the consultation and is repeated 4 weeks after consultation in which the treatment decision is made [17]. If an endovascular or open surgical treatment takes place within 4 weeks, decisional conflict is scored just before treatment. The decisional conflict in patients is scored using the 16-item Decisional Conflict Scale (DCS) [25].

Quality of life in patients is scored directly after consultation and again 6 weeks after treatment with the Short Form Health Survey (SF12) [26].

SDM as perceived by patients is scored directly after consultation using the SDM-Q-9 questionnaire, the 3-item CollaboRATE questionnaire, and the one-question Control Preference Scale and Control Perception Scale (CPS) [27–29]. The Control Preference Scale documents the desired amount of patient involvement and is scored before the consultation. The Control Perception Scale assesses the actually perceived amount of patient involvement and is scored after the consultation.

SDM as perceived by vascular surgeons is scored after the consultation using the SDM-Q-DOC questionnaire and the Control Perception Scale [29, 30].

The treatment decided upon is derived from the audio recording of the consultation. The actually received treatment is obtained from the participating vascular surgeon or centre.

The extent in which DSTs are implemented is determined by scoring the number of times a specific tool is used as recorded by the audio recording (consultation card and decision cards). Successful use of the decision aid is defined as completion of the decision aid by the patient. Completion and time to complete is recorded automatically when patients access the decision aid via the provided link.

Process measures studied are the number and duration of consultations necessary to decide upon a treatment, as obtained from the audio recording(s) or from the participating vascular surgeon or centre.

All outcomes mentioned above are evaluated at the individual participant level.

### Participants’ timeline

**Fig. 2:**
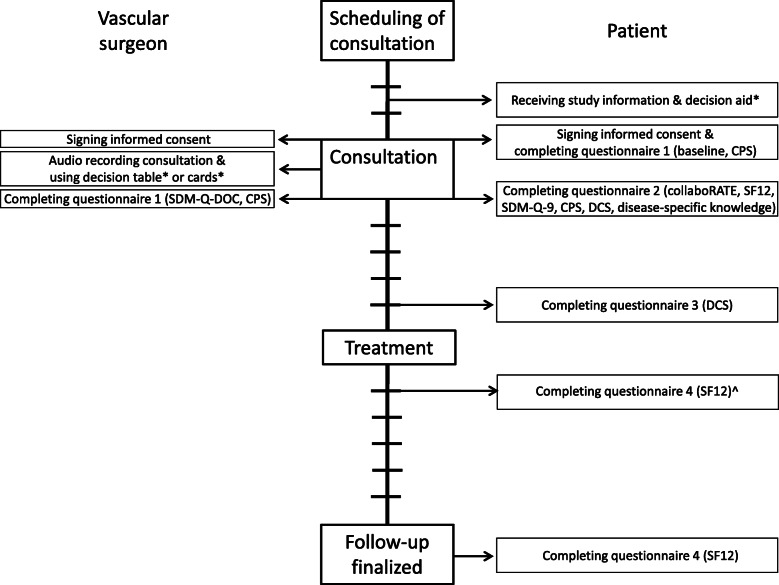
Participants’ timeline of actions during the trial. ┼: One week. *: Intervention group. ^: In case of conservative treatment. SF12: Short form health survey. CPS: Control preference or perception scale. DCS: Decisional conflict scale. SDM: Shared decision-making

Figure 2 provides an overview of the participants’ timeline. Patients in the intervention group receive the decision aid prior to the appointment at the outpatient clinic. Patient follow-up is finalised after the patient has completed the final questionnaire. Patients receive the final questionnaire 6 weeks after the treatment or, in case of conservative treatment, 6 weeks after the decision-making consultation.

### Sample size

Sample size calculations are based on a clinically relevant difference in the use of SDM during consultation before and after introduction of the DSTs. The systematic review of Couët et al. [31] found a mean increase from 23 (SD 14) to 34 (SD 8) of SDM scored with the 5-item OPTION instrument. An 11 out of 100 points increase of the 5-item OPTION instrument seems a meaningful improvement as it means an increase from a ‘minimal’ to a ‘moderate’ effort to involve patients in the decision-making process [31]. A larger increase would, of course, be even more clinically relevant and would require fewer patients, but this is less likely to reach. With a significance level of 0.05 and a power of 90%, a total sample size of 58 patients is required.

This number needs correction for the stepped-wedge design with cluster randomisation, as opposed to an individual patient randomisation. The total sample size from the power analysis is to be multiplied by the design effect for a stepped wedge trial. The pre-specified number of 5 steps (i.e. 6 time periods) and 5 clusters results in 58/(6*5) = 2 patients, per cluster per time period. Assuming an intermediate-level intra-cluster correlation of 0.01, the stepped wedge design effect is 1.944 [32]. Thus, the total sample size needed in this trial is 58 × 1.944 = 113 patients per disorder. Since four different vascular diseases are studied, there are actually four trials in one trial. Therefore, a multiplication of the total sample size by 4 is necessary, which leads to 452 patients. To adjust for a loss-to-follow-up of 10%, the study aims to include a total of 502 patients.

### Recruitment

All consecutive patients visiting the outpatient clinical of participating medical centres are screened for eligibility. Eligible patients are contacted by the researcher, nurse practitioner or surgeon and informed about the trial via the informed consent materials. The patient is given a minimum of 2 days to consider participation. Next, the patient is asked to participate in the study and to sign the informed consent form.

### Allocations

Participating medical centres are randomised into five different clusters, containing three centres. These clusters are again randomised every 2 months thereafter to decide which cluster is next to start applying the DSTs, as shown in Fig. 1. The researchers evaluate at each randomisation instance whether sufficient patients have been included in the trial. If inclusion rate falls behind, randomisation of the next cluster to use the DSTs is delayed for another 2 months. The researchers randomise the participating centres and clusters by drawing lots stating the name of a participating centre or cluster from an opaque container.

### Blinding

Due to the nature of this study it is not possible to blind patients or vascular surgeon, since they actively use the intervention. However, the cluster randomisation design does reduce potential contamination of information among the participating vascular surgeons. It is also not possible to blind the researchers scoring the five OPTION items on audio-recordings. The use of consultation cards and decision cards is audible and most vascular surgeons will inquire whether the patient has used the decision aid.

### Data collection methods

Trial data are obtained via questionnaires, audio recordings, the decision aid content management system, and participating vascular surgeons. Patients fill out the questionnaires either at their medical centre, at home via email, or on paper accompanied by a stamped self-addressed envelope.

### Data management

All obtained trial data are considered as confidential information and will not be distributed to third parties. Patient data are stored anonymously under a code. Only the principal investigator or researchers authorised by the principal investigator have access to the key file.

### Statistical methods

Baseline characteristics are summarised using descriptive statistics. Unevenly distributed outcome measures are expressed as medians and inter-quartile ranges. A differential effect among the four included vascular diseases is not expected, as the primary outcome is SDM. SDM is equally applicable to each of these diseases since multiple treatment options are available. Nevertheless, the sample size is sufficient to analyse the effect on SDM for each disease separately.

Differences in mean scores of the 5-item OPTION instrument between consultations in which usual care is provided (control group) and the consultations in which DSTs are used (intervention group), are analysed using the Student t-test with Satterthwaite correction for unequal variances. ANCOVA is applied to correct for possible baseline differences in patients before and after the introduction of the DSTs.

Differences in (semi-)continuous variables between the usual care group and the DSTs group (e.g. Likert scales and quality of life scores) are analysed by means of the Student t-test or the non-parametric Mann-Whitney U test, depending on the normality of their distribution. Percentages are compared using a Chi-square test (e.g. for the final treatment choice). In particular, before-after differences in DCS at 4 weeks are analysed after correcting for differences between the groups in baseline DCS. Logistic regression analysis is used to determine the individual effect of the different DSTs on our primary outcome. Statistical analyses will be conducted using IBM SPSS version 24 (IBM, Armonk, NY, USA).

### Monitoring

Previous studies show that SDM has no adverse effects on health outcomes [1, 5, 9–12]. Therefore, no monitoring committee was assembled.

### Research ethics approval

The Medical Ethics Review Committee of the Academic Medical Center, Amsterdam, reviewed and approved version 2.0, dated 27 September 2017, of our trial protocol and written informed consent procedure.

### Protocol amendments

The researchers will notify participating centres, the Medical Ethics Review Committee and the Netherlands trial registry if protocol amendments arise.

### Consent or assent

Vascular surgeons, physician assistants, nurse practitioners or researchers inform patients eligible for participation about the OVIDIUS trial. Patients receive this information verbally and on paper, via the informed consent materials.

### Access to data

All obtained trial data are considered confidential information and will not be distributed to third parties. Participating vascular surgeons are able to obtain anonymous patient data only on request and when presenting with a relevant question.

### Ancillary and post-trial care

After the trial, the DSTs will be made publicly available via the patient advocacy group (Dutch patient organization for people with cardiovascular diseases) and the Dutch professional society (Dutch Society for Vascular Surgery).

### Dissemination policy

No restrictions have been placed on the publication of trial outcomes. The trial results are to be published in relevant scientific journals, preferably as open-access to ensure high accessibility. Authorship is granted based on the International Committee of Medical Journal Editors guidelines. The authors also plan to present the trial outcomes at national and international conferences.

## Discussion

Vascular surgery is pre-eminently a field in which SDM can enhance quality of care by incorporating patients’ preferences in the decision-making process, since there is commonly a conservative, endovascular or open surgical treatment available for most vascular surgical disorders. Unfortunately, the use of SDM is still limited amongst vascular surgeons. We therefore developed DSTs to assist vascular surgeons and their patients in shared decision-making. The OVIDIUS trial was designed to implement these DSTs into the vascular surgical consultation room and to study their effect on SDM.

Strengths of the OVIDIUS trial are first of all that both the patient advocacy group and the Dutch Society for Vascular Surgery were involved in the development of the DSTs, which is a prerequisite for a nationwide implementation of these DSTs to foster SDM. Second, 15 medical centres throughout the Netherlands participate in this study, including university and general hospitals, thus reducing selection bias by including uncomplicated cases only. Third, the stepped-wedge cluster-randomised study design minimizes the influence of any intercurrent changes in local protocols on the clinical outcomes during the trial period and it ensures that at the end of the study all participating centres have implemented at least some of the DSTs and thereby a certain level of SDM.

Limitations of the OVIDIUS trial are, first a potential inclusion bias of patients. Patients who actively want to be involved in the decision-making process may be more willing to participate, whereas patient who prefer the surgeon to make the decision are less inclined to participate. That is why the preferred decision-making strategy is assessed via the CPS questionnaire prior to consultation. Second, the trial is powered for four different diseases, even though their incidences differ. Hence, the researchers must closely monitor the inclusion rates of these different diseases and take appropriate action when one disease is included much more frequently than another.

The OVIDIUS trial will evaluate whether the developed DSTs can be implemented in clinical practice and whether they actually improve the level of SDM by showing an improvement of the 5-item OPTION score measured on audio recordings made in the vascular surgical consultation room. Perhaps even more important is the renewed attention that our trial generates regarding the benefits of using SDM amongst vascular surgeons. Future researchers and developers of DSTs can use this study protocol to set up their own trial for the evaluation and implementation of newly developed DSTs.

## Supplementary information

**Additional file 1.** SPIRIT 2013 Checklist: Recommended items to address in a clinical trial protocol and related documents*.

## Data Availability

The decision support tools and datasets used and/or analysed during the OVIDIUS study are available from the corresponding author on reasonable request.

## Abbreviations

AAA: Abdominal aortic aneurysm
AMC: Academic Medical Center
CAD: Carotid artery disease
CPS: Control preference/perception scale
DCS: Decisional conflict scale
DST: Decision support tools
IC: Intermittent claudication
OPTION: Observing patient involvement
OVIDIUS: Operative Vascular Intervention Decision-making Improvement Using SDM-tools
SD: Standard deviation
SDM: Shared decision-making
SDM-Q-9: Shared decision-making patient questionnaire
SDM-Q-DOC: Shared decision-making physician questionnaire
SF12: Short form health survey
SPIRIT: Standard Protocol Items: Recommendations for Interventional Trials
VV: Varicose veins
